# Effects on the Thermo-Mechanical and Interfacial Performance of Newly Developed PI-Sized Carbon Fiber–Polyether Ether Ketone Composites: Experiments and Molecular Dynamics Simulations

**DOI:** 10.3390/polym15071646

**Published:** 2023-03-25

**Authors:** Hana Jung, Kwak Jin Bae, Yuna Oh, Jeong-Un Jin, Nam-Ho You, Jaesang Yu

**Affiliations:** Composite Materials Application Research Center, Institute of Advanced Composite Materials, Korea Institute of Science and Technology, Chudong-ro 92, Bongdong-eup, Wanju-gun 55324, Republic of Korea

**Keywords:** carbon fiber, polyimide, sizing agent, interfacial adhesion, thermo-mechanical properties, molecular dynamics simulations

## Abstract

In this study, polyether ether ketone (PEEK) composites reinforced with newly developed water-dispersible polyimide (PI)-sized carbon fibers (CFs) were developed to enhance the effects of the interfacial interaction between PI-sized CFs and a PEEK polymer on their thermo-mechanical properties. The PI sizing layers on these CFs may be induced to interact vigorously with the p-phenylene groups of PEEK polymer chains because of increased electron affinity. Therefore, these PI-sized CFs are effective for improving the interfacial adhesion of PEEK composites. PEEK composites were reinforced with C-CFs, de-CFs, and PI-sized CFs. The PI-sized CFs were prepared by spin-coating a water-dispersible PAS suspension onto the de-CFs, followed by heat treatment for imidization. The composites were cured using a compression molding machine at a constant temperature and pressure. Atomic force and scanning electron microscopy observations of the structures and morphologies of the carbon fiber surfaces verified the improvement of their thermo-mechanical properties. Molecular dynamics simulations were used to investigate the effects of PI sizing agents on the stronger interfacial interaction energy between the PI-sized CFs and the PEEK polymer. These results suggest that optimal amounts of PI sizing agents increased the interfacial properties between the CFs and the PEEK polymer.

## 1. Introduction

In recent years, polyether ether ketone (PEEK) polymer composites reinforced with carbon fibers (CFs) have been widely used in high-performance applications, such as in the aerospace and automobile fields, due to their high heat resistance, very light weight, and great mechanical strength [1,2,3,4,5,6,7,8,9,10,11,12,13,14]. However, PEEK polymer has a high melting temperature of around 343 °C, which is constrained during the manufacturing process to improve the physical properties of the resulting composites [15,16,17,18,19]. Therefore, it was necessary to develop a sizing agent to protect the CFs with better thermal stability to increase the interfacial performance between the carbon fibers and matrix, which is an important factor in the load transfer of composites.

A variety of treatment methods (e.g., plasma, synthesis, and inducing functional groups of sizing agents on the fiber surfaces) have been studied [20,21,22,23,24,25,26,27,28,29]. Naito [30] demonstrated an increase in the tensile properties of composites reinforced with carbon nanotube (CNT)-grafted and polyimide (PI)-coated CFs. Hassan et al. [31] proposed that a loosely packed CNT network using PI could improve the interfacial adhesion between CFs and matrix due to enhanced CF wettability, polarity, and roughness. As a compatibilizer, PI exhibits a relatively high molecular weight in the interface regions as a result of a synergetic effect between PI and CNT. However, because PI is hard to dissolve, it requires a large amount of a strong organic solvent (such as N-Methyl-2-pyrrolidone) and limits the enhancement of interfacial adhesion [32,33,34,35]. Therefore, there is a need for novel research to create PI with high heat resistance for use as a sizing agent for carbon fibers.

Molecular dynamics (MD) simulations are attractive for predicting the effects of surface characteristics on the physical properties of polymer-based composites reinforced with carbon material [36,37,38]. It is possible to examine the thermal behavior of polymer-based composites by calculating the movement of molecules over time, which cannot be confirmed via experiments. Jiao et al. [39] and Jin et al. [40] studied the interfacial properties between polymers and sized-, functionalized-carbon materials using MD simulations. However, these results are not considered to prove enhanced interfacial properties without molecular mobility, which is significant in the thermal behavior of composites.

In this study, the thermo-mechanical properties and optimal sizing agent content of CF/PEEK composites with sizing agents, which can be applied even at the high processing temperature of PEEK polymers, were investigated. The aqueous PI sizing agent effects on the structure, morphology, interface performance, and thermo-mechanical properties of CF/PEEK composites were analyzed in detail to prove the comprehensive contributions of environmentally friendly modifications using various surface analysis techniques (Fourier-transform infrared spectroscopy (FT-IR), nuclear magnetic resonance (NMR), atomic force microscopy (AFM), and field-emission scanning electron microscopy (FE-SEM)) and physical estimates (interlaminar shear strength (ILSS), and dynamic mechanical analyzer (DMA) and thermogravimetric analyzers (TGA) characteristics). MD simulations were performed to analyze the interfacial characterization and thermal behavior according to the effect of the PI sizing agent between the CFs and the PEEK polymer. The calculated results can validate the experiments of the CF/PEEK composites.

## 2. Experiments

### 2.1. Materials

Pyromellitic dianhydride (PMDA, >99%), 4,4′-oxydianiline (ODA, >98%), and 4-hydroxy-1-methylpiperidine (HMP, >98%) were purchased from Tokyo Chemical Industry (Tokyo, Japan) to synthesize the poly (amic acid) salt (PAS). The N-Methyl-2-pyrrolidinone (NMP) was obtained from Sigma-Aldrich (St. Louis, MO, USA). A CF plain-weave fabric (C120-3K, plain, HD FIBER Co., Ltd., Yangsan, KR) was used as reinforcement for the composites. C120-3K has a density of 1.78 g∙cm^−3^, a weight of 203 g∙m^−2^, and a thickness of 0.25 mm. The amorphous PEEK film (APTIV 2000, Victrex, Co., Ltd., Lancashire, UK) was used as a composite matrix with high heat resistance. The PEEK film has a density of 1.26 g∙cm^−3^, a tensile modulus of 1.80 GPa, a thickness of 16 μm, and a glass transition and melting temperature of 143 and 343 °C, respectively.

### 2.2. Preparation of Poly (Amic Acid) Salt (PAS) as a PI Sizing Agent

**Figure 1:**
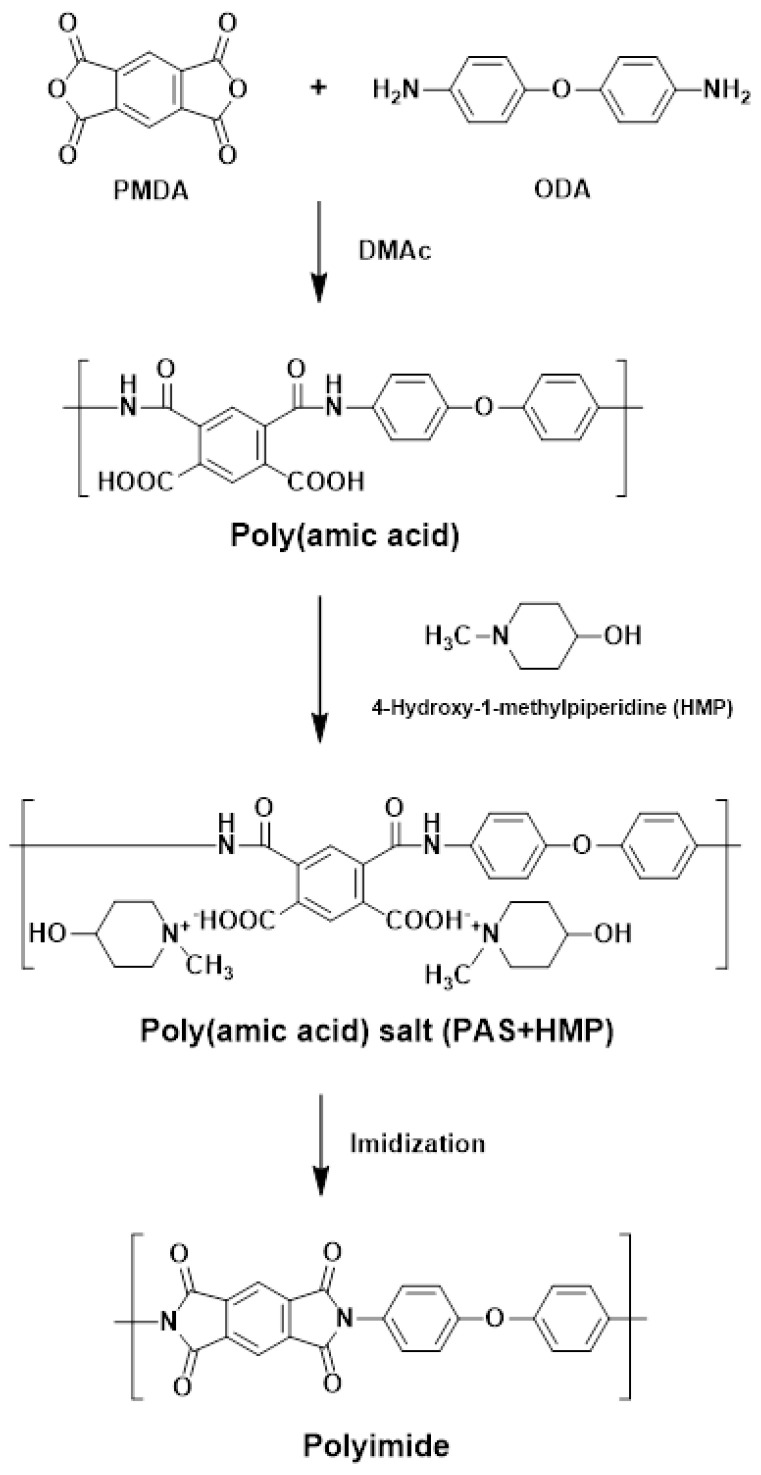
Synthetic route for PAS.

The polyimide (PI) was prepared from poly (amic acid) (PAA), a precursor, using a two-step reaction. The first step was the formation of the PAA solution via a nucleophilic substitution reaction of the monomer in a step-growth polymerization reaction. The second step was the dehydration and cyclization of the synthesized PAA solution with thermal treatment over 200 °C resulting in the final PI sizing agent. The synthetic process of the PAS powder was as follows (Figure 1). An amount of 0.15 mol of ODA was dissolved in 1200 mL of NMP at room temperature. Then, 0.15 mol of PMDA was added to the ODA solution, and the resulting mixture was stirred for 24 h under N_2_ gas. This resulted in the formation of a PAA solution. Then, 0.3 mol of HMP was added to the PAA solution and stirred for 1 h. The mixture was then precipitated in acetone, and the resulting product was filtered, washed, and dried under vacuum at room temperature to obtain a PAS powder [15].

### 2.3. Fabrication of Water-Dispersible PI-Sized CF/PEEK Composites

**Figure 2:**
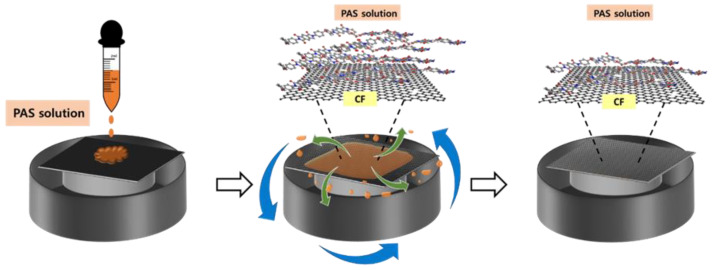
Schematic diagram of the process for surface treatment of carbon fibers using the PAS solution.

Suspensions were created to deploy the sizing agent onto the carbon fiber surfaces using the PAS powder in distilled water. The water-dispersible PAS powder was heated in a water bath at 60 °C for 4 h to obtain the PI sizing agent, which was homogenized on a hot plate by stirring it with a magnetic bar. Sizing agents were prepared with PI contents of 0.5, 1.0, and 1.5 wt% compared with the CFs. The commercial carbon fabric (C-CF) was immersed in acetone at room temperature for 2 h to remove epoxy groups from the sizing agent. Then, the carbon fabric was cleaned in acetone in 2 steps (10 min each) and dried in a vacuum oven at 40 °C. The de-sized carbon fabric (de-CF) was modified by coating it with the prepared PAS suspensions using a spin coater at 800 rpm for 30 s. The PAS-modified CFs with different contents of 0.5, 1.0, and 1.5 wt% were dried in an oven at 40 °C for 12 h. For the imidization of the PAS-modified CF, heat treatment was carried out in an oven at 250 °C for 1 h. An illustration of the surface treatment of the CFs with the PI sizing agent is shown in Figure 2. In this study, the laminate was composed of 25 layers of carbon fiber fabric and 150 plies of PEEK film. PEEK composites were reinforced with C-CF, de-CF, and PI-sized CF. The laminate was preheated using a compression-molding machine at a constant temperature of 380 °C for 10 min without pressure. After the preheating process, the temperature was maintained at 380 °C with a pressure of 10 MPa for 20 min. The cooling step was performed using a water-cooling system with a rate of −10 °C∙min^−1^. The total weight fraction of the carbon fibers was about 58 wt%.

### 2.4. Characterizations

Nuclear magnetic resonance (NMR) spectra were determined with an Agilent 600 MHz Premium COMPACT spectrometer at 600 MHz for ^1^H in dimethyl sulfoxide-*d*_6_ (DMSO-*d*_6_), using tetramethylsilane (TMS) as an internal standard. The attenuated total reflection–Fourier-transform infrared (ATR-FT-IR) spectra were obtained with a Nicolet IS10 with 32 scans per spectrum at a 2 cm^−1^ resolution. Atomic force microscopy (AFM) was conducted with a peak force tapping mode system (multimode-8, BRUKER Co., Billerica, MA, USA) to measure the surface roughness of the carbon fibers. Field-emission scanning electron microscopy (FE-SEM Verios 460L, FEI Corp., Hillsboro, OR, USA) with energy-dispersive X-ray spectroscopy (EDX) was performed to characterize the surface morphologies of the carbon fibers as well as the fracture surfaces of the composites reinforced with carbon fibers. The interlaminar shear strength (ILSS) of the composites was measured using the short-beam shear test as three-point bending according to the ASTM D 2344 standard (5569, Instron, Norwood, MA, USA) [41]. Specimens with the dimensions 28.2 mm × 9.4 mm × 4.7 mm were tested at a cross-head speed rate of 1 mm∙min^−1^. The short-beam strength (*F*^sbs^) was calculated from the equation *F*^sbs^ = 0.75 P_b_/*bh*, where Pb represents the maximum load, b is the width, and h is the thickness of the specimen. The sample results were estimated as an average value from more than five specimens. The thermo-mechanical properties of the composites were obtained using a dynamic mechanical analyzer (DMA Q800, TA Instruments, New Castle, DE, USA) with a dual-cantilever mode at 1 Hz from 30 °C to 300 °C and a heating rate of 3 °C∙min^−1^, according to the ASTM 4065-01 standard [42]. The thermal stability of the carbon fibers was analyzed using thermogravimetric-differential thermal analysis (TG-DTA Analyzer SDT Q600, WATERS, Milford, MA, USA) with a temperature range of 25 °C to 800 °C with a heating rate of 10 °C min^−1^ under nitrogen gas. 

### 2.5. Molecular Dynamics Modeling

**Figure 3:**
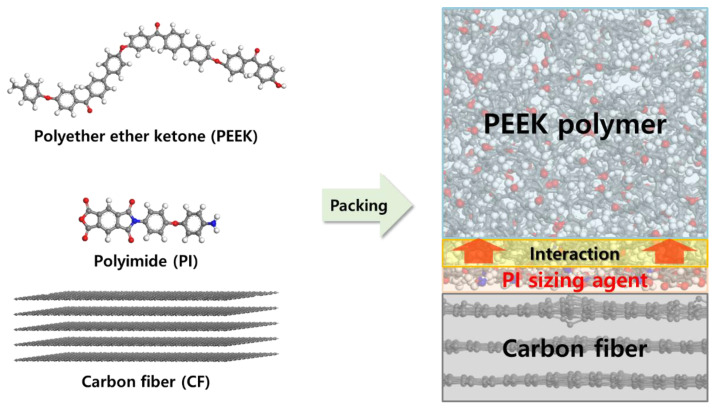
Molecular structures and composite models in the MD simulations.

MD simulations were performed to investigate the effect of the PI sizing agent on the interface between the CFs and PEEK polymer using Materials Studio 2017 software. The simulation model consisted of five graphene sheets, PI molecular chains, and PEEK molecular chains for modeling (Figure 3). The PI molecular chains with 0.0, 0.3, 1.0, 2.0, and 5.0 wt% compared with the carbon fibers were placed on the top surfaces of the graphene sheets. The simulation of the system based on a constant number, volume, and temperature (NVT) ensemble was performed to equilibrate all the models with a time step of 1.0 fs for 300 ps at 300 K. The simulation of the system based on a constant number, pressure, and temperature (NPT) ensemble was performed to further relax the internal stresses of the simulation models with an additional annealing process. All the models were heated from 300 K to 700 K at 50 K intervals and cooled down to 300 K at equal intervals. Each step was performed with a time step of 1.0 fs during 300 ps. The density of the equilibrated CF/PEEK models was 1.54 g∙cm^−3^, which was similar to the experimental result of 1.55 g∙cm^−3^. The dimension of the CF/PEEK composite model was 48.2 Å (*x*) × 49.9 Å (*y*) × 53.1 Å (*z*) in the periodic boundary condition. All the MD simulations were performed using the condensed-phase-optimized molecular potentials for the atomistic simulation studies (COMPASS ΙΙ) force field [43], which has been widely used to describe the interaction between carbon materials and polymers. 

The mean square displacement (MSD) can be used to characterize the speed at which particles move and quantify the mobility of molecules according to temperature. The MSD could be determined in the temperature range from 250 K to 500 K during the NPT ensemble. The MSD analysis can be calculated using the following equation:

(1) $$\mathrm{MSD}=\frac{1}{N}\overset{N}{\underset{i=1}{\sum }}{\left|x^{\left(i\right)}\left(t\right)-x^{\left(i\right)}\left(0\right)\right|}^{2}$$

where N is the number of particles to be averaged, $x^{\left(i\right)}\left(0\right)$ is the reference position of the i-th particle, and $x^{\left(i\right)}\left(t\right)$ is the position of the i-th particle at time t.

### 2.6. Interfacial Characterization

The interaction energy of the CFs with the PEEK polymer was calculated using

(2) $$\Delta E\;\left(kcal/mol\right)=E_{total}-\left(E_{CF}+E_{polymer}\right)$$

where ΔE is the interaction energy at the interface between the CFs and the PEEK polymer, $E_{total}$ represents to the total potential energy of the composite, and $E_{CF}$ and $E_{polymer}$ are the potential energy of the PI-sized CFs and PEEK polymer, respectively. The interfacial shear strength (ISS, *τ*) between the PI-sized CFs and the PEEK polymer was calculated using

(3) $$\tau \;\left(MPa\right)=\frac{\Delta E}{WL^{2}}$$

where τ is the ISS, W is the width of the CF, L represents the length of the CF, and ΔE corresponds to the difference in the interaction energy.

## 3. Results and Discussion

### 3.1. Synthesis and Characterization of PAS

**Figure 4:**
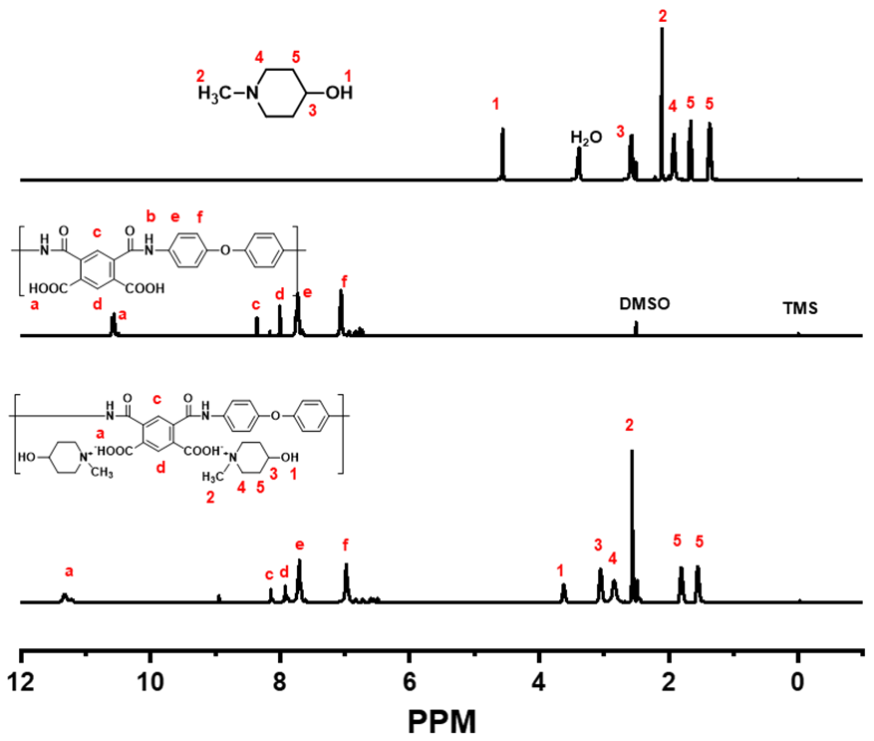
^1^H NMR spectra of HMP, PAA, and PAS.

The ^1^H NMR spectrum of PAS could be assigned by comparison between the ^1^H NMR spectra of HMP and PAA, as shown in Figure 4. The marked numbers and letters in the Figure 4 were noticed each molecular group and spectrum peak. The peaks at 2.56 ppm in the ^1^H NMR spectrum of HMP were related to the 3 protons of the CH_3_ groups. The protons in PAA could be confirmed from the peaks at 6–9 ppm in the ^1^H NMR spectrum of PAA. Those peaks were also found in the ^1^H NMR spectrum of PAS. Moreover, the 6 peaks at 1.5–4 ppm in the ^1^H NMR spectrum of PAS that appeared after the reaction of PAA with HMP were related to a proton on the piperidine ring of HMP. The proton of the CH_3_ group in HMP was de-shielded by a proton of the COOH group in PAA. 

**Figure 5:**
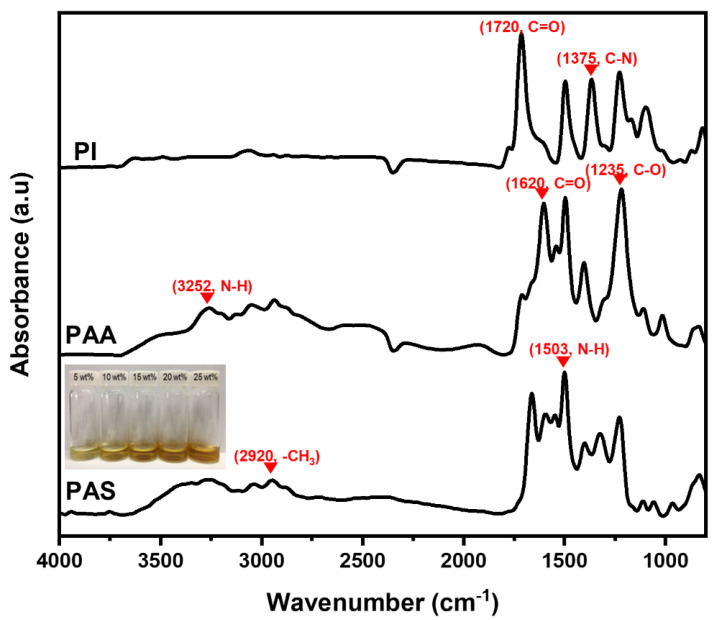
FT-IR spectra of PI, PAA, and PAS.

Figure 5 shows the FT-IR spectra of PI, PAA, and PAS. The FT-IR spectrum of PAA shows N-H stretching at 3252 cm^−1^, C=O stretching at 1620 cm^−1^, N-H stretching at 1503 cm^−1^, and C-O stretching at 1235 cm^−1^. The FT-IR spectrum of PAS shows new bands at 3380 cm^−1^ and 2920 cm^−1^, corresponding to the O-H and -CH_3_ bonds of the HMP groups in PAS. The FT-IR spectrum of PAS, which can also be observed in the FT-IR spectrum of PAA, indicates that PAS was completely synthesized, as shown in Figure 5. The FT-IR spectra of PAA, PAS, and PI were used to verify the imidization of PAS to obtain cured PI. This was conducted via the observation of the disappearance of the peak at 1620 cm^−1^ and the growth of a peak at 1720 cm^−1^. In Figure 5, the photographic image represents the aqueous PAS solution in water with an increase in concentration according to our previous study [15]. The synthesized PAS powder was readily available to the water-dispersible sizing agent without organic solvents.

### 3.2. Surface Morphology Analysis of the PI-Sized CFs

**Figure 6:**
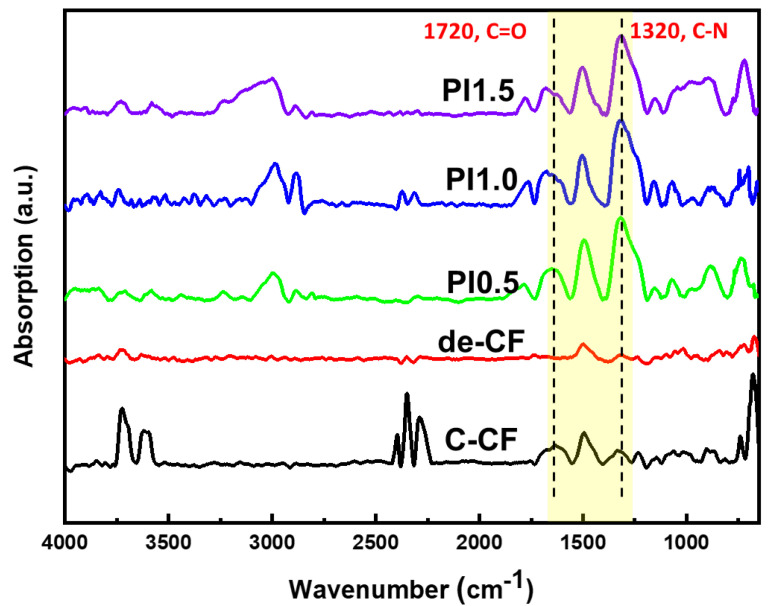
FT-IR spectra of C-CFs and de-CFs as well as 0.5, 1.0, and 1.5 wt% PI-sized CFs.

The surface structures of the CFs were characterized using FT-IR, AFM, and FE-SEM observations. The FT-IR spectra of the C-CFs, de-CFs, and PI-sized CFs with contents of 0.5, 1.0, and 1.5 wt% were combined in Figure 6. The spectrum of the de-CFs was relatively smoother than that of the C-CFs due to the removal of epoxy groups and any other residues. A sharp carboxylic acid absorption peak (C=O stretching) in all the PI-sized CF samples was observed near 1700 cm^−1^. The addition of PAS molecules onto the CF surfaces reveals N-H stretching. This was assigned to 1320 cm^−1^, which was not displayed in the C-CFs. It could be determined that the carbon fiber surfaces were successfully modified with the cured PI from the newly synthesized water-dispersible PAS, which led to a more effective increase in the carbon fiber surface polarity and interfacial adhesion between the matrix and fibers.

**Figure 7:**
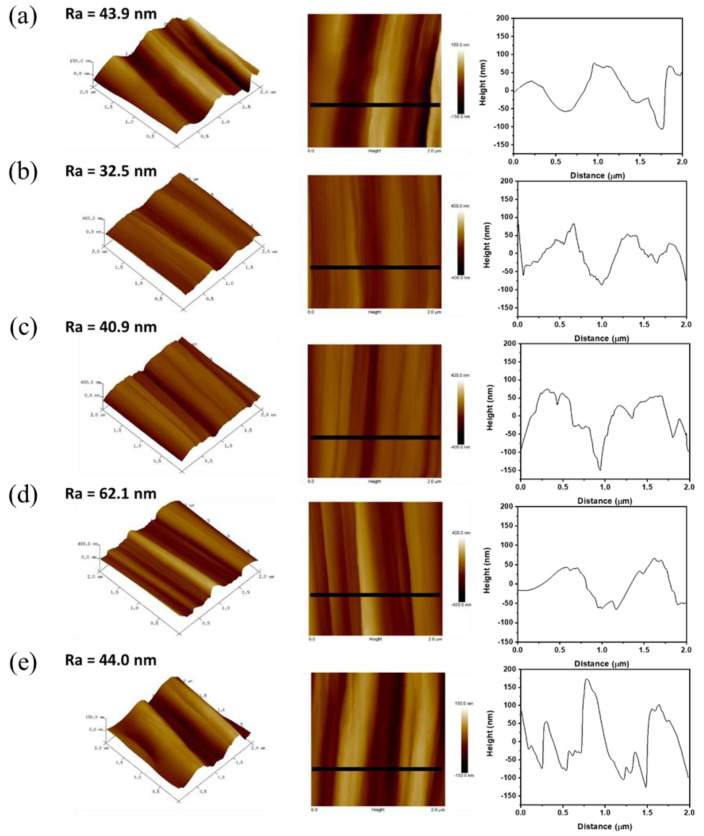
AFM images and height profiles for (**a**) C-CFs and (**b**) de-CFs, as well as (**c**) PI 0.5 wt%, (**d**) PI 1.0 wt%, and (**e**) PI 1.5 wt% of PI-sized CFs.

The 2D and 3D surface topographies of the de-CFs, C-CFs, and PI-sized CFs with different contents of PI sizing agents were compared, as shown in Figure 7. Increased surface roughness was observed on the PI-sized CF surfaces compared with those of the de-CFs. The average roughness (R_a_) of the PI-sized CFs with contents of 0.5, 1.0, and 1.5 wt% (40.9, 62.1, and 44.0 nm, respectively) was greater than that of the de-CFs (32.5 nm). This increased roughness led to the enhancement of mechanical interlocking between the surface of the PI-sized CFs and the PEEK polymer [44]. However, in the case of the PI 1.5 wt% PI-sized CFs, the variation in height became relatively high (from −150 nm to 170 nm) due to an excessive amount of coating, as shown in Figure 7e [45,46]. This agglomerated non-uniform sizing layer between the carbon fibers and the matrix can lead to a decrease in interfacial adhesion.

**Figure 8:**
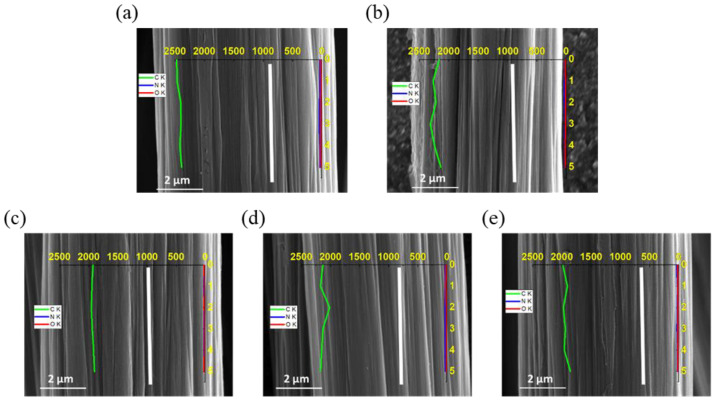
FE-SEM images and SEM-EDX line spectra of elemental carbon (green line), nitrogen (blue line), and oxygen (red line) in (**a**) C-CFs and (**b**) de-CFs, as well as (**c**) PI 0.5 wt%, (**d**) PI 1.0 wt%, and (**e**) PI 1.5 wt% PI-sized CFs.

Observations using SEM-EDX were performed to analyze the compositional elements of reactive PI groups on the carbon fiber surfaces. Figure 8 shows the PI line profile of the EDX spectra across the carbon fiber surface along the scanline. The graph demonstrates the intensity of elemental carbon (C), nitrogen (N), and oxygen (O) with respect to distance along the fiber surface. The elements N and O had relatively uniform low concentrations over all of the carbon fiber surfaces. However, the C line profile shows an obvious decrease according to the increase in the content of the PI sizing agent, as shown in Figure 8a,e. This result was ascribed to a PI sizing layer containing uniformly attached N or O compared with the C-CFs or de-CFs. Increasing the amount of N or O greatly affects the interfacial adhesion between CFs and a PEEK polymer due to solubility with higher ionization [47].

### 3.3. Thermo-Mechanical Properties of the PI-Sized CF/PEEK Composites

**Figure 9:**
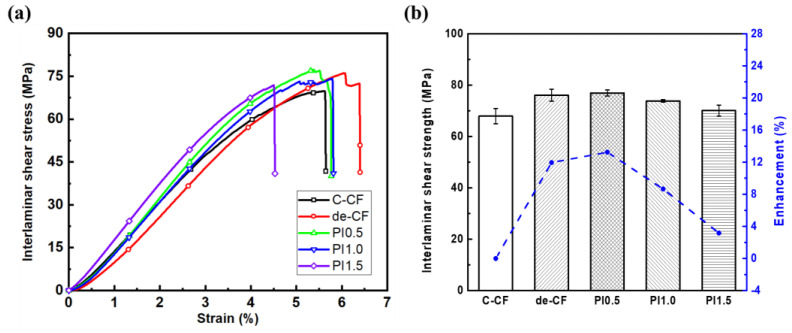
Interlaminar shear properties of composites reinforced with C-CFs and de-CFs, as well as PI 0.5 wt%, PI 1.0 wt%, and PI 1.5 wt% PI-sized CFs. (**a**) stress–strain curves; (**b**) interlaminar shear strengths and enhancements.

The ILSS results were analyzed to investigate the characteristics of the interface between the CFs and the PEEK polymer, with or without the PI sizing agent, as shown in Figure 9. The short-beam strength for the PI 0.5-, 1.0-, and 1.5-sized CF-reinforced PEEK composites was 76.9, 73.8, and 71.4 MPa, respectively. The ILSSs of all PI-sized CFs and de-CF/PEEK composites were higher (up to 13.2%) than those of the C-CF/PEEK composites with the commercial sizing agents. The structure of the aromatic PI synthesized from the PAA salt of the PMDA/ODA type shows a charge-transfer interaction consisting of electron donors (including nitrogen groups) and electron acceptors (including carbonyl groups). This charge-transfer complex was attributed to the intramolecular forces between PI molecules, as well as to the intermolecular forces between PI molecules and PEEK polymer chains. These properties were supported by the strong molecular attraction due to the formation of robust polymer chains. With all these reactive groups, it was possible for them to attach layer by layer. Therefore, polyimides may not only form well-stacked sizing layers on the surfaces of carbon fibers but can also interact well with the p-phenylene groups of PEEK polymer chains [31]. This leads to an enhanced potential for the PI sizing agent to cause improved interfacial adhesion between the CFs and the PEEK polymer. The fiber surfaces of the de-CFs could not be protected from abrasion due to the lack of the sizing agent. In addition, the de-CFs were difficult to handle due to low cohesion. Therefore, the de-CFs are limited in fiber processing and production and composite material fabrication. Therefore, it is necessary for the treatment with a sizing agent to be appropriate to preserve the carbon fibers [48,49]. Figure 9 shows that the 0.5 wt% content of the PI sizing agent improved the short-beam strength of the CF/PEEK composites by approximately 13%. The commercial sizing agent is generally known to achieve approximately 1% coating on the surfaces of carbon fibers. This result demonstrates that composites reinforced with the newly developed water-dispersible PI have competitiveness for their efficient application to high-performance structures.

**Figure 10:**
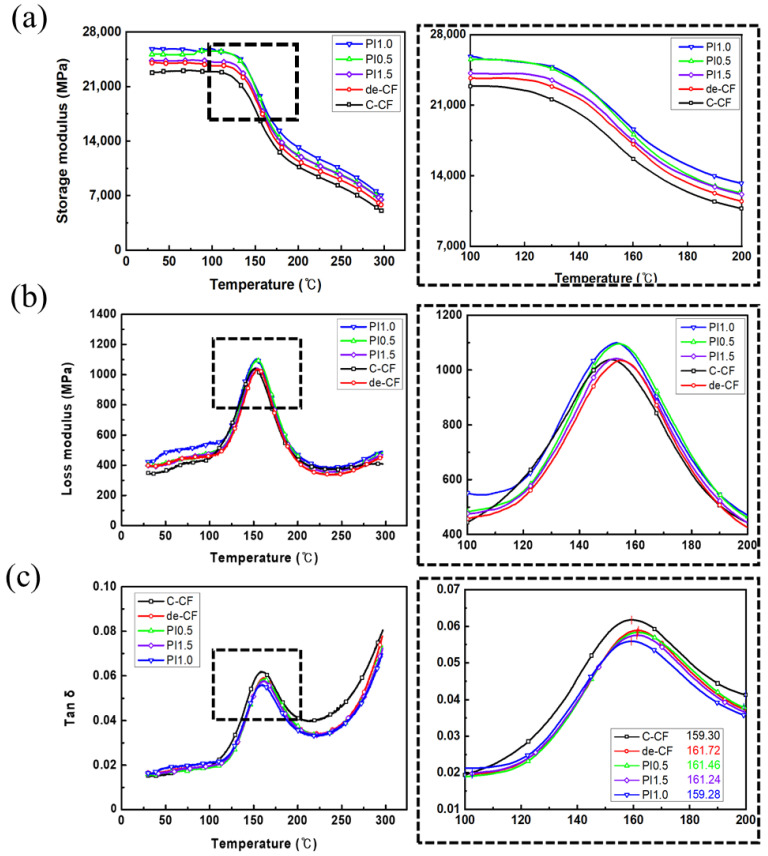
(**a**) Storage moduli, (**b**) loss moduli, and (**c**) tan δ curves for the composites reinforced with C-CFs and de-CFs, as well as PI 0.5 wt%, PI 1.0 wt%, and PI 1.5 wt% of PI-sized CFs.

**Figure 11:**
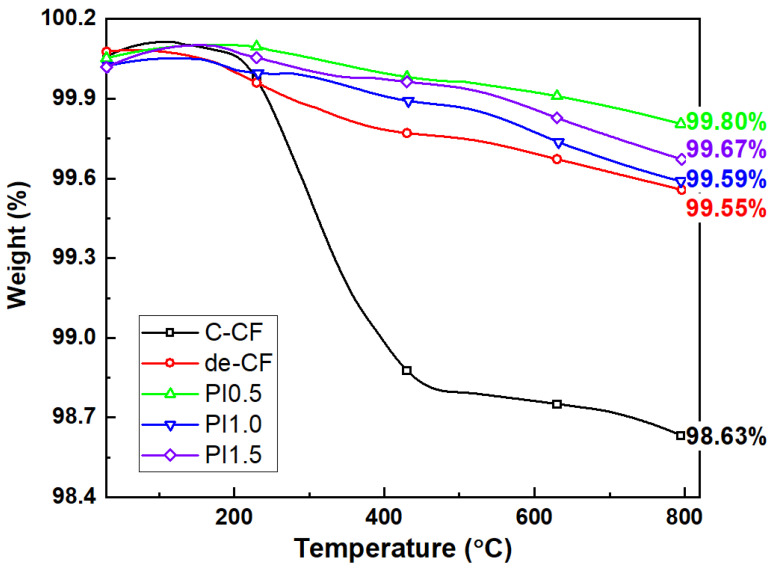
TGA curves of C-CFs, de-CFs, PI 0.5 wt%, PI 1.0 wt%, and PI 1.5 wt% sized CFs.

The DMA results on the viscoelastic properties of the composites reinforced with the C-CFs, de-CFs, and PI-sized CFs are shown in Figure 10. These results indicate that the complex modulus between the polymers can be affected by a wide variety of chain motions over a range of temperatures. Figure 10a shows a comparative increase in stored energy for all of the composites containing PI-sized CFs compared with the composites containing commercial epoxy-sized CFs. The storage moduli of the composites with 0.5, 1.0, and 1.5 wt% PI-sized CFs were 23,076, 24,440, and 23,724 MPa, respectively. In the case of the 1.0 wt% PI-sized CFs, the rate of increase in the storage modulus was approximately 11.6% compared with that of the C-CF/PEEK composites. As mentioned above, this is because the interfacial adhesion between the carbon fibers and the PEEK polymer was improved due to enhanced intermolecular attraction by adding the PI sizing agent. The effect of the PI sizing agent was demonstrated while ensuring excellent thermal stability at a high temperature. In addition, the increase in the loss modulus, which indicates energy dissipated as heat from the composites, approaches that of its viscous property. The composite reinforced with 1.0 wt% PI-sized CFs is distinctively represented in Figure 10b. This phenomenon means that the intermolecular force associated with the interfacial interaction between the PI sizing agent and the PEEK polymer was even greater than the intramolecular force between the CFs and the PI sizing agent of the PI-Sized CF/PEEK composites. This result indicates that the surface modification of the carbon fibers could be determined by the interfacial failure attributed to the physical properties of the composites. In addition, Figure 10c shows that the glass transition temperatures of the PI-Sized CF/PEEK composites were increased in comparison with the C-CF/PEEK composites. Among the content levels of the PI sizing agent, the appropriate amount on the surface of a CF is 0.5 wt% for the storage modulus as well as the damping factor (tan δ). This means that the thermal resistance to molecular mobility in the composites with the PI sizing agent was enhanced in comparison with the C-CFs or de-CF/PEEK composites [34]. Therefore, the effective incorporation of PI on the CFs was important to improve the thermo-mechanical properties of the PEEK composites reinforced with CFs. As shown in Figure 10c, the measured width from the tan δ curve for the composite reinforced C-CFs is remarkably wide, which indicates a greater distribution of the segment lengths related to molecular mobility than in the PI-Sized CF/PEEK composites. Moreover, the intensity of the tan δ curve for the composite-reinforced C-CFs presents a greater increase in the mobility of the relaxing segments of the polymer compared with that of the composite-reinforced PI-sized CFs. This refers to the weak interaction bonding resulting from poor inter–intramolecular forces between the C-CFs and the PEEK polymer with increasing temperature. These results suggest that the interfacial interaction between CFs and PEEK can be affected by the PI sizing agent on the surfaces of the carbon fibers. This effect was attributed to the significantly enhanced interfacial adhesion of the composites. The effect of the sizing agent with high heat resistance was the remarkable thermal stability of the CF/PEEK composites. In addition, the thermal stability of the PI-sized CFs with high heat resistance was compared with that of the C-CFs, as shown in Figure 11. The residual weights of the composites with C-CFs, de-CFs, PI0.5, PI1.0, and PI1.5 were 98.63, 99.55, 99.80, 99.59, and 99.67%, respectively. In the case of the PI-sized CFs with contents of 0.5, 1.0, and 1.5 wt%, there was no weight loss over the temperature range of 25 °C to 800 °C. The difference in the weight loss ratio between the PI-sized CFs and C-CFs was definitely observed depending on the absence of the polyimide sizing agent. Therefore, it was maintainable to protect the surfaces of the carbon fibers with the residual amount of the polyimide sizing agent after decomposition at a high temperature.

### 3.4. Fracture Characteristics of the CF/PEEK Composites

**Figure 12:**
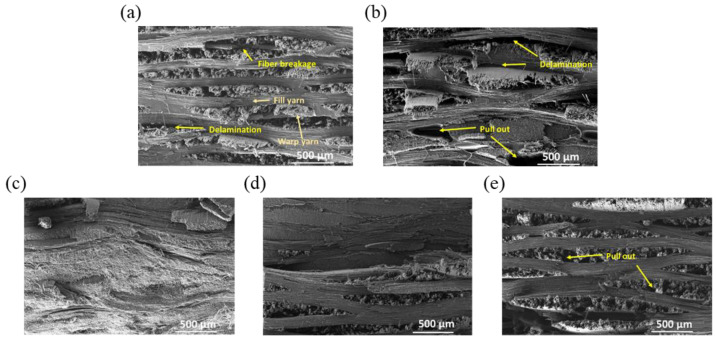
FE-SEM images of fracture surfaces on composites reinforced with (**a**) C-CFs and (**b**) de-CFs, as well as (**c**) PI 0.5 wt%, (**d**) PI 1.0 wt%, and (**e**) PI 1.5 wt% of the PI-sized CFs.

Figure 12 shows SEM images of the fracture surfaces on the composites reinforced with carbon fibers after the ILSS test. The fracture surfaces of the composites reinforced with C-CFs and de-CFs are relatively rough compared with those of the composites reinforced with PI-sized CFs at all content levels. Damaged fibers in the fill direction were rumpled, and pull-out of fibers were observed in the warp direction (Figure 12a,b). In addition, while delamination between the C-CFs or de-CFs and PEEK matrix was observed, the fracture surfaces of the PI 0.5, 1.0, and 1.5 wt% PEEK composites are rather smooth and tightly embedded between the CFs and the PEEK matrix. This phenomenon confirms that the interfacial adhesion was enhanced by adding the PI sizing agent to the carbon fibers [50]. The PI sizing agent was protected due to its high heat resistance during high-temperature manufacturing processes, which allowed it to withstand the effective stress transfer of the carbon fiber. The PI-sized CFs played an important role in improving the interfacial performance of these PEEK composites.

### 3.5. Molecular Dynamics of the CF/PEEK Composite

**Figure 13:**
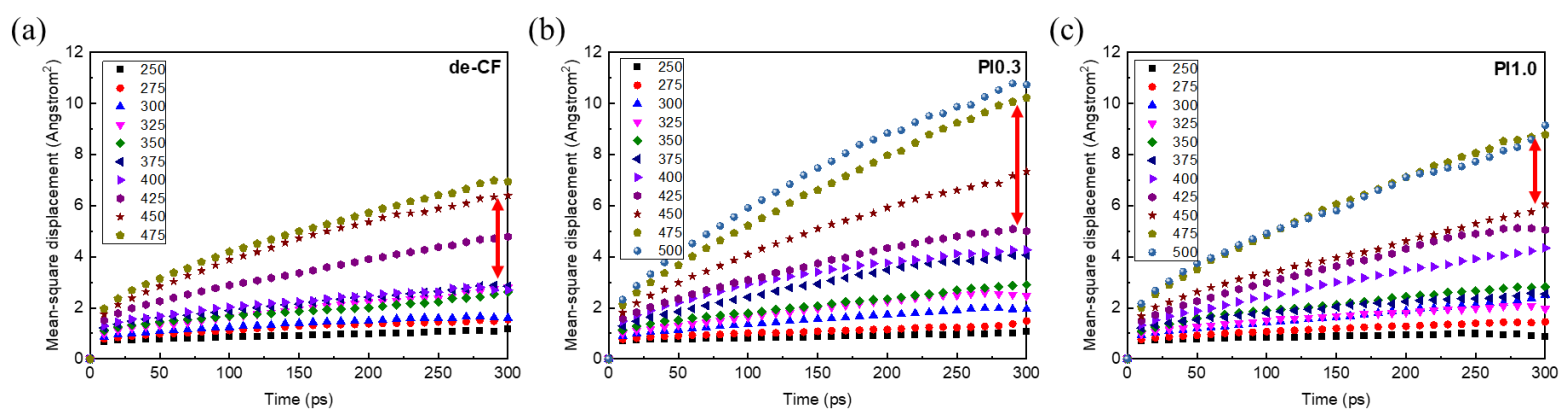
Mean square displacement (MSD) results: (**a**) de-CFs, (**b**) PI0.3, and (**c**) PI1.0.

MSD analysis can derive an estimation of the parameters of molecular movements, such as the diffusion coefficient. When the model systems reached the phase transition region, the diffusion level increased sharply, which can determine the glass transition temperature. The temperatures of the phase transition regions of the composite models were increased, and are marked with a red arrow in Figure 13. The temperatures of the phase transition regions of the composite models with de-CFs, PI0.3, and PI1.0 were 400–450 K, 425–475 K, and 450–475 K, respectively. The de-CFs (Figure 13a) show a relatively low temperature range compared with the PI-sized composites. This result shows that the PI sizing agent increased the glass transition temperature.

**Figure 14:**
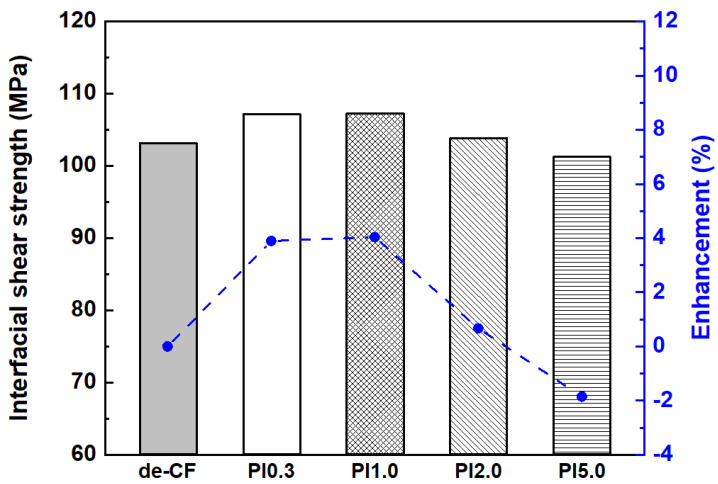
Interfacial shear strength of PEEK composites reinforced with de-CFs as well as PI 0.3 wt%, PI 1.0 wt%, PI 2.0 wt%, and PI 5.0 wt% sized CFs using MD simulation.

The interfacial characteristics between the de-CFs and the PEEK polymer were calculated using MD simulations. Table 1 shows the interaction energy (ΔE) between the CFs and the PEEK polymer. The negative interfacial energy means that the energy of adsorption of the PEEK molecules at the interface was strong [51,52]. Therefore, the high negative value indicates strong adhesion at the interface between the CFs and the PEEK polymer. The interaction energy of the de-CF/PEEK composites was −1788.79 kcal/mol. Those of the PI-Sized CF/PEEK composites with contents of PI 0.3, 1.0, 2.0, and 5.0 wt% were −1877.73, −1869.48, −1815.94, and −1767.29 kcal/mol, respectively. These results show that the interaction energies of the PI-Sized CF/PEEK composites with contents of PI 0.3, 1.0, and 2.0 wt% generally had a higher interaction energy than that of the de-CF/PEEK composite. In addition, the PI sizing agents also affected the interfacial shear strength (ISS), as shown in Figure 14. The interfacial shear strengths of the de-CFs, PI0.3, PI1.0, PI2.0, and PI5.0 were calculated to be 103.15, 107.17, 107.31, 103.84, and 101.24 MPa, respectively, obtained from Equation (3). The simulated interfacial shear strengths of the PI-Sized CF/PEEK composites, except for the PI5.0 model, were higher than that of the de-CF/PEEK composite. This result proves that the PI sizing agent affected the interfacial properties between the CFs and the PEEK polymer. In addition, the contents of 0.3 wt% and 1.0 wt% of the PI sizing agent on the CFs were the most effective for improving the interaction energy and interfacial shear strength. The results obtained from the MD simulations show similar trends to the experimental data.

## 4. Conclusions

In this study, the effects of water-dispersible PI-sized CFs on the thermo-mechanical properties of PEEK composites were investigated. The PMDA/ODA used for the PAA solution was successfully synthesized and thermally imidized to apply it as a sizing agent for the carbon fibers. The surfaces of the carbon fibers were modified by adding a PI sizing layer that led to improved interfacial adhesion between the CFs and PEEK matrix. The short-beam strengths and storage moduli of the PEEK composites reinforced with PI-sized CFs increased more than those of the composites reinforced with de-sized and commercial CFs. The surface roughness of the PI-sized CFs as indicated in the 2D and 3D topographies was greater than those of the C-CFs and de-CFs. This result shows that the CF surfaces were coated with the PI sizing agent. The fracture surfaces of the composites reinforced with PI-sized CFs exhibited greater smoothness and were more tightly embedded between the CFs and PEEK matrix than those of the composites reinforced with C-CFs and de-CFs. This was ascribed to enhanced mechanical interlocking between the PI-sized CFs and the PEEK polymer of the composites. These results suggest that the interfacial interaction between CFs and PEEK can be affected by a PI sizing agent on the surfaces of carbon fibers. This effect was attributed to the significantly enhanced interfacial adhesion of the composites even at a high temperature. MD simulations were performed to examine the effects of the PI sizing agents on the interfacial properties between the CFs and the PEEK polymer. The interfacial properties between the CFs and the PEEK polymer were most improved when the PI sizing agent with 0.3 wt% was applied to the surface of the carbon fibers. As a result, the newly developed PI sizing agent with high heat resistance can be used to improve the interfacial bonding properties and thermo-mechanical properties of CF/PEEK composites. The present study focused on improving the interfacial bonding properties and thermo-mechanical properties of CF/PEEK composites. PI-sized carbon nanomaterials with high-temperature resistance and high dispersion can be investigated to improve the thermo-mechanical properties of polymer-based composites in the future.

## Figures and Tables

**Table 1:** Interaction energy of CF/PEEK composites using MD simulation.

Sample (300 K)		$E_{total}\;(\mathrm{kcal}/\mathrm{mol})$	$E_{CF}\;(\mathrm{kcal}/\mathrm{mol})$	$E_{polymer}\;(\mathrm{kcal}/\mathrm{mol})$	$\Delta E_{\;}\;(\mathrm{kcal}/\mathrm{mol})$
de-CF/PEEK		243,256.35	229,233.60	15,811.53	−1788.79
PI-Sized CF/PEEK	0.3 wt%	243,991.70	229,315.41	16,554.02	−1877.73
	1.0 wt%	244,734.37	229,173.83	17,430.01	−1869.48
	2.0 wt%	242,697.81	229,127.90	15,385.85	−1815.94
	5.0 wt%	240,873.99	228,557.79	14,083.48	−1767.29

## Data Availability

The data presented in this study are available on request from the corresponding author.

## References

[1] Wang X.K., Huang Z.G., Lai M.L., Jiang L., Zhang Y., Zhou H.M. (2020). Highly enhancing the interfacial strength of CF/PEEK composites by introducing PAIK onto diazonium functionalized carbon fibers. Appl. Surf. Sci..

[2] Yan T.W., Yan F., Li S.Y., Li M., Liu Y., Zhang M.J., Jin L., Shang L., Liu L., Ao Y.H. (2020). Interfacial enhancement of CF/PEEK composites by modifying water-based PEEK-NH2 sizing agent. Compos. B Eng..

[3] Stepashkin A.A., Chukov D.I., Senatov F.S., Salimon A.I., Korsunsky A.M., Kaloshkin S.D. (2018). 3D-printed PEEK-carbon fiber (CF) composites: Structure and thermal properties. Compos. Sci. Technol..

[4] Yan M.X., Tian X.Y., Peng G., Li D.C., Zhang X.Y. (2018). High temperature rheological behavior and sintering kinetics of CF/PEEK composites during selective laser sintering. Compos. Sci. Technol..

[5] Pan L., Yapici U. (2016). A comparative study on mechanical properties of carbon fiber/PEEK composites. Adv. Compos. Mater..

[6] Sharma M., Bijwe J., Mader E., Kunze K. (2013). Strengthening of CF/PEEK interface to improve the tribological performance in low amplitude oscillating wear mode. Wear.

[7] Molazemhosseini A., Tourani H., Khavandi A., Yekta B.E. (2013). Tribological performance of PEEK based hybrid composites reinforced with short carbon fibers and nano-silica. Wear.

[8] Zhao W.Y., Yu R., Dong W.Y., Luan J.S., Wang G.B., Zhang H.B., Zhang M. (2021). The influence of long carbon fiber and its orientation on the properties of three-dimensional needle-punched CF/PEEK composites. Compos. Sci. Technol..

[9] Lessard H., Lebrun G., Benkaddour A., Pham X.T. (2015). Influence of process parameters on the thermo-stamping of a [0/90]_(12)_ carbon/polyether ether ketone laminate. Compos. Part. A Appl. Sci. Manuf..

[10] Vieille B., Albouy W., Taleb L. (2015). Influence of stamping on the compressive behavior and the damage mechanisms of C/PEEK laminates bolted joints under severe conditions. Compos. B Eng..

[11] Stokes-Griffin C.M., Compston P. (2015). The effect of processing temperature and placement rate on the short beam strength of carbon fibre-PEEK manufactured using a laser tape placement process. Compos. Part. A Appl. Sci. Manuf..

[12] Dworak M., Rudawski A., Markowski J., Blazewicz S. (2017). Dynamic mechanical properties of carbon fibre-reinforced PEEK composites in simulated body-fluid. Compos. Struct..

[13] Na R.Q., Liu J.Y., Wang G.B., Zhang S.L. (2018). Light weight and flexible poly (ether ether ketone) based composite film with excellent thermal stability and mechanical properties for wide-band electromagnetic interference shielding. RSC Adv..

[14] Yang Y.C., Wang T.J., Wang S.D., Cong X., Zhang S.L., Zhang M., Luan J.S., Wang G.B. (2020). Strong Interface Construction of Carbon Fiber-reinforced PEEK Composites: An Efficient Method for Modifying Carbon Fiber with Crystalline PEEK. Macromol. Rapid Commun..

[15] Jung H., Bae K.J., Jin J., Oh Y., Hong H., You N.H., Yu S.J. (2020). The effect of aqueous polyimide sizing agent on PEEK based carbon fiber composites using experimental techniques and molecular dynamics simulations. Func. Compos. Struct..

[16] Gao X.P., Huang Z.G., Zhou H.M., Li D.Q., Li Y., Wang Y.M. (2019). Higher mechanical performances of CF/PEEK composite laminates via reducing interlayer porosity based on the affinity of functional s-PEEK. Polym. Compos..

[17] Lu C.R., Xu N., Zheng T., Zhang X., Lv H.X., Lu X., Xiao L., Zhang D.X. (2019). The Optimization of Process Parameters and Characterization of High-Performance CF/PEEK Composites Prepared by Flexible CF/PEEK Plain Weave Fabrics. Polymers.

[18] Chang B.N., Li X.M., Parandoush P., Ruan S.L., Shen C.Y., Lin D. (2020). Additive manufacturing of continuous carbon fiber reinforced poly-ether-ether-ketone with ultrahigh mechanical properties. Polym. Test..

[19] Yao S.S., Jin F.L., Rhee K.Y., Hui D., Park S.J. (2018). Recent advances in carbon-fiber-reinforced thermoplastic composites: A review. Compos. B Eng..

[20] Hassan E.A.M., Yang L.L., Elagib T.H.H., Ge D.T., Lv X.W., Zhou J.F., Yu M.H., Zhu S. (2019). Synergistic effect of hydrogen bonding and pi-pi stacking in interface of CF/PEEK composites. Compos. B Eng..

[21] Guo L.H., Zhang G., Wang D.A., Zhao F.Y., Wang T.M., Wang Q.H. (2017). Significance of combined functional nanoparticles for enhancing tribological performance of PEEK reinforced with carbon fibers. Compos. Part. A Appl. Sci. Manuf..

[22] Zhang G. (2010). Structure-Tribological Property Relationship of Nanoparticles and Short Carbon Fibers Reinforced PEEK Hybrid Composites. J. Polym. Sci. B Pol. Phys..

[23] Liu H.S., Zhao Y., Li N., Zhao X.R., Han X., Li S., Lu W.K., Wang K., Du S.Y. (2019). Enhanced interfacial strength of carbon fiber/PEEK composites using a facile approach via PEI&ZIF-67 synergistic modification. J. Mater. Res. Technol..

[24] Chen J.L., Wang K., Zhao Y. (2018). Enhanced interfacial interactions of carbon fiber reinforced PEEK composites by regulating PEI and graphene oxide complex sizing at the interface. Compos. Sci. Technol..

[25] Moosburger-Will J., Bauer M., Laukmanis E., Horny R., Wetjen D., Manske T., Schmidt-Stein F., Topker J., Horn S. (2018). Interaction between carbon fibers and polymer sizing: Influence of fiber surface chemistry and sizing reactivity. Appl. Surf. Sci..

[26] Jang J., Kim H. (1997). Improvement of carbon fiber/PEEK hybrid fabric composites using plasma treatment. Polym. Compos..

[27] Mao J.H., Pan Y.S., Ding J. (2019). Tensile mechanical characteristics of CF/PEEK biocomposites with different surface modifications. Micro Nano Lett..

[28] Zabihi O., Ahmadi M., Li Q., Shafei S., Huson M.G., Naebe M. (2017). Carbon fibre surface modification using functionalized nanoclay: A hierarchical interphase for fibre-reinforced polymer composites. Compos. Sci. Technol..

[29] Li Q.X., Church J.S., Naebe M., Fox B.L. (2016). Interfacial characterization and reinforcing mechanism of novel carbon nanotube-Carbon fibre hybrid composites. Carbon.

[30] Naito K. (2014). Tensile Properties of Polyimide Composites Incorporating Carbon Nanotubes-Grafted and Polyimide-Coated Carbon Fibers. J. Mater. Eng. Perform..

[31] Hassan E.A.M., Ge D.T., Zhu S., Yang L.L., Zhou J.F., Yu M.H. (2019). Enhancing CF/PEEK composites by CF decoration with polyimide and loosely-packed CNT arrays. Compos. Part. A Appl. Sci. Manuf..

[32] Kim H., Ku B.C., Goh M., Ko H.C., Ando S., You N.H. (2019). Synergistic Effect of Sulfur and Chalcogen Atoms on the Enhanced Refractive Indices of Polyimides in the Visible and Near-Infrared Regions. Macromolecules.

[33] He S.Q., Zhang S.C., Lu C.X., Wu G.P., Yang Y., An F., Guo J.H., Li H. (2011). Polyimide nano-coating on carbon fibers by electrophoretic deposition. Colloids Surf. A Physicochem. Eng. Asp..

[34] Wang T., Jiao Y.S., Mi Z.M., Li J.T., Wang D.R.N., Zhao X.G., Zhou H.W., Chen C.H. (2020). PEEK composites with polyimide sizing SCF as reinforcement: Preparation, characterization, and mechanical properties. High Perform. Polym..

[35] Naganuma T., Naito K., Yang J.M. (2011). High-temperature vapor deposition polymerization polyimide coating for elimination of surface nano-flaws in high-strength carbon fiber. Carbon.

[36] Jung H., Choi H.K., Oh Y., Hong H., Yu J. (2020). Enhancement of thermomechanical stability for nanocomposites containing plasma treated carbon nanotubes with an experimental study and molecular dynamics simulations. Sci. Rep..

[37] Jung H., Choi H.K., Kim S., Lee H.S., Kim Y., Yu J. (2017). The influence of N-doping types for carbon nanotube reinforced epoxy composites: A combined experimental study and molecular dynamics simulation. Compos. Part A Appl. Sci. Manuf. S..

[38] Li Y.L., Wang S.J., Wang Q. (2017). A molecular dynamics simulation study on enhancement of mechanical and tribological properties of polymer composites by introduction of graphene. Carbon.

[39] Jiao W.W., Hou C.L., Zhang X.H., Liu W.B. (2021). Molecular dynamics simulation of the influence of sizing agent on the interfacial properties of sized carbon fiber/vinyl ester resin composite modified by self-migration method. Compos. Interfaces.

[40] Jin Y.K., Duan F.L., Mu X.J. (2016). Functionalization enhancement on interfacial shear strength between graphene and polyethylene. Appl. Surf. Sci..

[41] (2013). Standard Test Method for Short-Beam Strength of Polymer Matrix Composite Materials and Their Laminates; Annual Book of ASTM Standards.

[42] (2001). Standard Practice for Plastics: Dynamic Mechanical Properties: Determination and Report of Procedures; Annual Book of ASTM Standards.

[43] Sun H. (1998). COMPASS: An ab initio force-field optimized for condensed-phase applications-Overview with details on alkane and benzene compounds. J. Phys. Chem. B.

[44] Yao Y., Chen S.H. (2013). The effects of fiber’s surface roughness on the mechanical properties of fiber-reinforced polymer composites. J. Compos. Mater..

[45] Liu H.S., Zhao Y., Li N., Li S., Li X.K., Liu Z.W., Cheng S., Wang K., Du S.Y. (2021). Effect of polyetherimide sizing on surface properties of carbon fiber and interfacial strength of carbon fiber/polyetheretherketone composites. Polym. Compos..

[46] Kong D.C., Yang M.H., Zhang X.S., Du Z.C., Fu Q., Gao X.Q., Gong J.W. (2021). Control of Polymer Properties by Entanglement: A Review. Macromol. Mater. Eng..

[47] Raghavendran V.K., Drzal L.T., Askeland P. (2002). Effect of surface oxygen content and roughness on interfacial adhesion in carbon fiber-polycarbonate composites. J. Adhes. Sci. Technol..

[48] Zheng H., Zhang W.J., Li B.W., Zhu J.J., Wang C.H., Song G.J., Wu G.S., Yang X.P., Huang Y.D., Ma L.C. (2022). Recent advances of interphases in carbon fiber-reinforced polymer composites: A review. Compos. B Eng..

[49] Tang L.G., Kardos J.L. (1997). A review of methods for improving the interfacial adhesion between carbon fiber and polymer matrix. Polym. Compos..

[50] Hwang D., Lee S.G., Cho D. (2021). Dual-Sizing Effects of Carbon Fiber on the Thermal, Mechanical, and Impact Properties of Carbon Fiber/ABS Composites. Polymers.

[51] Sun S.Q., Chen S.H., Weng X.Z., Shan F., Hu S.Q. (2019). Effect of Carbon Nanotube Addition on the Interfacial Adhesion between Graphene and Epoxy: A Molecular Dynamics Simulation. Polymers.

[52] Zuo Z., Liang L.F., Bao Q.Q., Yan P.T., Jin X., Yang Y.L. (2021). Molecular Dynamics Calculation on the Adhesive Interaction Between the Polytetrafluoroethylene Transfer Film and Iron Surface. Front. Chem..

